# Parental executive functions and motivations unveil variations in young children's screen media use

**DOI:** 10.1186/s41155-024-00289-z

**Published:** 2024-06-07

**Authors:** Paulo Guirro Laurence, Matheus de Melo Rodrigues, Maria Carolina Brito Locatti Tannus, Elisa Macedo Dekaney, Elizeu Coutinho Macedo

**Affiliations:** 1 Social and Cognitive Neuroscience Laboratory, Center for Health and Biological Sciences, Mackenzie Presbyterian University, Rua Piaui, nº 181, 10Th Floor, São Paulo, 01241-001 Brazil; 2 Developmental Disorders Program, Center for Health and Biological Sciences, Mackenzie Presbyterian University, São Paulo, Brazil; 3 College of Visual & Performing Arts, Syracuse University, Syracuse, NY USA; 4 National Institute of Science and Technology On Social and Affective Neuroscience, São Paulo, Brazil

**Keywords:** Screen media usage, Cognitive abilities, Parental motivations, Motivations for media use, Socioeconomic status

## Abstract

**Background:**

The increased screen media use among children aged 3 to 5, particularly in the post-COVID era, is concerning. Despite several organizations' recommendation of a one-hour screen limit for young children, actual usage often exceeds this guideline. Objective: This study explored the influence of parental characteristics such as self-efficacy, motivation, socioeconomic status, and cognitive abilities on children's screen time habits.

**Methods:**

Employing a feature selection model, 251 caregivers answered an online survey, presenting data from themselves and on-screen usage for 126 girls and 125 boys. We found that the caregiver’s executive functions, including cognitive flexibility, initiation, task monitoring, and material organization, significantly impact children’s screen time. Results: Our analysis highlighted the vital role of caregivers’ self-efficacy in moderating children's screen usage. Family net income, children's age and gender, and motivations related to children's desires and behavioral control were also significant contributors to usage patterns.

**Conclusion:**

This study offers insights into interventions and effective parenting strategies in the digital age, highlighting the importance of addressing socio-demographic factors in understanding this complex issue.

## Introduction

Screen media use by young children (3 to 5 years old) has been surging in recent years, and the global COVID-19 pandemic seems to have accelerated this trend (Bergmann et al., 2022). Multiple organizations have made recommendations for children's screen time usage. For example, the American Academy of Pediatrics (AAP) recommended no screen time for children between 18 and 24 months, excluding video chatting, while children aged between 2 and 5 should limit their screen media time to one hour per day (AAP, 2016). The World Health Organization (WHO) recommended that children under five limit screen media usage to an hour (WHO, 2019). However, on average, children aged 0 to 8 spend 2 1/2 h on screen media daily (Rideout & Robb, 2020). The disconnection between recommended guidelines and actual screen time usage underscores the urgent need for a comprehensive examination of this phenomenon. Although screen media usage can prompt cognitive development, it can also lead to behavior problems, anxiety, and depression (Stiglic & Viner, 2019).

Studies have explored the link between early screen exposure and later developmental outcomes, including academic performance, social skills, and mental health. Some research suggests that prolonged screen time during early childhood may lead to challenges in academic readiness (Christakis, 2009). Additionally, there is growing concern about the impact of screen media on social and emotional development, with potential connections to mental health issues (Primack et al., 2009).

Screen media usage can be affected by several factors, but caregivers' behaviors and cognitive abilities play a significant role because they act as gatekeepers of their children's screen media usage (Swider-Cios et al., 2023). Parents’ socioeconomic status (SES) and self-efficacy can affect children's screen media time, with higher SES negatively correlated with screen media time and higher parental self-efficacy positively correlated (Kieslinger et al., 2021). Guardians' motivation is also a factor given that higher noneducational media usage was linked to children’s asking to use screen-based media, children's leisure, and for providing time for guardians to complete their chores (Cingel & Krcmar, 2013). Furthermore, although parents may be interested in following the guidelines defined by organizations such as the AAP and WHO, they may not always be able to follow such recommendations (Wilson & Gross, 2018). In this case, Executive Functions (EF) play a significant role given that parents must control, organize, and prioritize certain aspects of their children's routines.

EFs are cognitive processes that act as a central command system for the human brain, governing a wide range of higher-order functions. They are often conceptualized using the Diamond (2013) model, which comprises several interrelated components. Inhibition, the first facet of EFs, involves the ability to suppress impulsive behaviors, thoughts, or responses, playing a crucial role in regulating screen media usage in both children and parents. Cognitive flexibility, the second component, enables individuals to adapt to changing situations and switch between tasks or rules, mirroring the adaptability required when managing screen time rules and content. Working memory, the third facet, involves temporarily holding and manipulating information in mind, which is indispensable when monitoring and implementing screen time guidelines. The EFs have also been related to everyday life (Roth et al., 2013). In this sense, several components have been identified as EFs, namely: self/emotional control, which relates to managing emotions and impulses, vital for parents when faced with children's screen-related requests; initiation, which governs the ability to begin tasks without external prodding, necessary for denying children screen-related requests; task monitoring, which involves overseeing ongoing activities and detecting errors, mirroring the need for parents to supervise their children's screen interactions; organization of materials, which is the ability to effectively arrange and select appropriate materials, aligning with the role parents play in curating suitable screen content for their children. EFs, as delineated by the Diamond model, are fundamental cognitive processes that underpin decision-making, self-regulation, and adaptability in various aspects of life, including the intricate realm of screen media usage (Bustamante et al., 2023; Diamond, 2013; Roth et al., 2013). It is possible to assume that guardians with different levels of EFs will have different approaches in how they mediate their children's screen usage.

Although EFs seem to be a relevant component for parents to mediate their children's screen media usage behaviors, this issue requires further investigation. Therefore, this study aimed to examine the relationship among parents’ EFs, motivation for their children's screen media usage, and children's screen media usage.

The study aligns with recent discourse on real-world data, emphasizing the significance of collecting diverse data sources, including electronic medical records, claims databases, patient-generated data, and surveys. Through rigorous analytics, real-world evidence is generated, offering distinct advantages over traditional healthcare assessments (Magalhães et al., 2022). In this context, the study focuses on capturing data related to various screen behaviors in children and their parents to provide new insights into the health implications for families.

Furthermore, in the context of modern daily life, a profound understanding of the screen media usage patterns exhibited by children and parents is imperative for the development of effective interventions. The third wave of cognitive and behavioral psychological interventions has played a transformative role in shaping the landscape of digital mental health for both children and parents. Diverging from traditional therapeutic modalities, these interventions incorporate advanced methodologies, including mindfulness-based techniques, acceptance and commitment therapy (ACT), and dialectical behavior therapy (DBT). Demonstrated efficacy, as supported by studies (Brigden et al., 2019; Hayes & Hofmann, 2021), positions these digital interventions as interactive tools capable of enhancing various cognitive, affective, attentional, self-regulatory, motivational, and behavioral aspects for both children and parents. The integration of these innovative approaches within digital mental health platforms establishes a comprehensive and accessible support system for families, with the overarching goal of fostering mental well-being and fortifying parent–child relationships within the framework of the digital age (Borghouts et al., 2021). Therefore, an in-depth exploration of the intricate relationships between parental EFs, motivations guiding their children's screen media usage, and the actual screen media usage by children holds the promise of yielding valuable insights within the context of these evolving intervention paradigms.

## Methods

### Participants

Through an online survey via Qualtrics, we collected self-reported data from parents, guardians, and caregivers regarding their SES, self-efficacy, EFs, motivation for their children’s screen media usage, and screen media usage patterns of their children. The survey was disseminated through multiple social media channels affiliated with the laboratory and the university, with participation being entirely voluntary and without any monetary compensation. Upon completion of the survey, participants were granted access to their instrument results and offered explanations for their scores. Given the comprehensive nature of the questionnaires, it is reasonable to assume that not all guardians had equal opportunities to complete the survey, leading to varying levels of engagement among respondents. Consequently, parents, guardians, and caregivers with more available time may have been more likely to fully participate in the questionnaire, introducing a potential source of bias in our data collection process.

Of the 702 surveys collected, 251 were fully completed and used in our analysis, reporting screen usage of 126 girls and 125 boys, with a mean age of 4.03 (SD = 0.79). Data collection occurred between March 2023 and August 2023. The University ethics committee approved the study (CAAE: 65318022.1.0000.0084), and all participants gave written consent. Descriptive information of our sample is shown in Table 1.

**Table 1:** Descriptive of sample socio-demographic information

Information	Characteristics	n	%	Mean (SD)	Min	Max
Parent	Age			34.13 (6.82)	18	59
	Gender					
	*Female*	236	94.0			
	*Male*	14	5.6			
	*Other/Non-disclosed*	1	0.4			
	Kinship with the child					
	*Mother*	226	90.0			
	*Father*	12	4.8			
	*Stepmother*	1	0.4			
	*Other/Non-disclosed*	12	4.8			
	Race					
	*White*	181	72.1			
	*Brown*	51	20.3			
	*Black*	11	4.4			
	*Asian*	8	3.2			
	Type of occupation					
	*Working outside of home*	98	39.0			
	*Working part-time from home*	51	20.3			
	*Working from home*	37	14.7			
	*Unemployed*	48	19.1			
	*Other/Non-disclosed*	17	6.8			
	Level of education					
	*Elementary school*	2	0.8			
	*High school*	33	13.1			
	*College*	84	33.5			
	*Post-grad specialization*	132	52.6			
	Net household income^a^					
	*Until 1 minimum wage*	12	4.8			
	*1–3 minimum wages*	35	13.9			
	*3–6 minimum wages*	52	20.7			
	*6–9 minimum wages*	41	16.3			
	*9–12 minimum wages*	38	15.1			
	*12–15 minimum wages*	17	6.8			
	*15–20 minimum wages*	22	8.8			
	*More than 20 minimum wages*	34	13.5			
	Number of people in the household			3.63 (0.88)	2	7
	*2*	9	3.6			
	*3*	119	47.8			
	*4*	86	34.5			
	*5*	27	10.8			
	*6*	6	2.4			
	*7*	2	0.8			
Child	Age			4.03 (0.79)	3	5
	Gender					
	*Girl*	125	49.8			
	Boy	126	50.2			
	Race					
	*White*	190	75.7			
	*Brown*	45	17.9			
	*Black*	7	2.8			
	*Asian*	9	3.6			
	Access to a daycare center or school					
	*Yes, full-time*	87	34.7			
	*Yes, part-time*	141	56.2			
	*No*	23	9.2			

^a^The minimum wage refers to Brazil's minimum wage. In 2023, the Brazilian minimum wage was 1320 BRL per month, and the mean USD to BRL conversion in the study's period was ~ 5.04. Therefore, the minimum Brazilian salary was ~ 261.90 USD per month

### Instruments

#### ScreenQ

The Screen-based media use in young children questionnaire (ScreenQ) is a self-report questionnaire with 15 questions about young children's screen media use. Parents and guardians give the report, and it evaluates four domains of screen-based media use in accordance with the AAP: access to screens, frequency of use, media content, and caregiver–child co-viewing. Each item score varies from 0 to 2. Higher scores indicate more screen-based media use against recommendations (Hutton et al., 2020). In our sample, the ScreenQ presented acceptable reliability (α = 0.74).

#### BRIEF-A

The Behavior Rating Inventory of Executive Function-Adult Version (BRIEF-A) is a self-report instrument with 75 questions about executive functioning in everyday life with a 3-level Likert scale for answers. It measures several EF indexes, such as inhibition (9 questions, e.g., “I have trouble waiting my turn”), cognitive flexibility (6 questions, e.g., “I am upset by unexpected changes in my routine”), self-monitoring (10 questions, e.g., “I overreact even to small problems”), self/emotional control (6 questions, e.g., “I don't realize when I make others feel bad or angry until it's too late”), initiation (7 questions, e.g., “I have difficulty starting tasks”), working memory (8 questions, e.g., “I have difficulty doing more than one activity at the same time”), planning (10 questions, e.g., “I have difficulty organizing my work”), task monitoring (6 questions, e.g., “I misjudge how difficult or easy a task will be”), and material organization (8 questions, e.g., “I can't organize myself”). It also contains 5 questions for validity (e.g., “I have trouble counting to three”). Each question can have a score from 0 to 2. Higher scores in the indexes suggest executive dysfunction (Roth et al., 2013). In this study, the BRIEF-A presented excellent internal consistency (α = 0.96).

#### Self-efficacy question

To assess the self-efficacy of guardians, parents, and caregivers regarding their children's screen-media usage, we asked them the following question: “How sure are you that you could say ‘no’ to your child’s request to perform activities that involve screen use (TV, videos, apps, video games)?”. The answers options (and scores) were not confident (1), a little confident (2), confident (3), very confident (4), and extremely confident (5).

#### PMSE-Q

The Parental Motivation for Screen Exposure Questionnaire (PMSE-Q) is an instrument that evaluates different motivations for children's use of screen-based media. It was elaborated based on Cingel and Krcmar's (2013) and Rideout and Robb's (2020) work. It is composed of 20 questions divided into four factors: Parental needs – when the use of screens is motivated by guardians doing different activities while they distract their children with screen-based media (e.g., “I let the child use electronic devices so that I can have free time”); Educational purposes – when the use of screens is motivated by educational purposes (e.g., “I let the child use electronic devices because I believe it helps him to develop his reasoning and language”); Children's desire – when the screen usage is motivated by the child’s desire, misbehaving if not offered screen-based media, or by family routine (e.g., “I let the child use electronic devices because he throws a tantrum if I do not offer electronic devices”); and Behavioral control – when the screen usage is motivated by rewarding certain behaviors of the children (e.g., “I let the child use electronic devices as a reward if he behaves well”). Answers (and scores) are: It is never a motivation for me (0); Sometimes it is a motivation for me (1); It is often a motivation for me (2); It is always a motivation for me (3). Higher scores indicate that the factor is one of the primary sources of motivation to let the child use screen-based media. In a Brazilian sample, the full instrument demonstrated robust internal consistency with a Cronbach's alpha of 0.93, while the specific factors within the instrument also exhibited strong reliability: parental needs (0.88), educational purposes (0.92), children's desire (0.87), and behavioral control (0.75) (Campos, 2022). These findings affirm the instrument's reliability and the stability of its individual factors in the context of this study.

#### Sociodemographic questionnaire

We made a sociodemographic questionnaire exclusively for this study to access information from the guardians and the children. This questionnaire asked about respondents' ages and their children, gender, and race. We also asked for family SES information, such as guardians’ occupations and education level, household population, daycare center or school access, and net household income.

### Data analysis

First, we analyzed the distribution of our variables. Since all presented a distribution similar to normal, we calculated multiple Pearson correlation coefficients between the ScreenQ total score, Parent Self-Efficacy, PMSE-Q factors, and BRIEF-A indexes. We sought to understand the relationship between the numerical variables for conducting regression models.

To understand the relationship among guardians’ EFs, motivation for their children's screen media usage, and young children's screen media usage, we employed a feature selection model, the Least Absolute Shrinkage and Selection Operator (LASSO) regression, which allowed us to identify the features related to our target variable (i.e., young children's screen media usage, measured by the ScreenQ) by shrinking their coefficients through the L1- regularization. This procedure penalizes the betas, and coefficients that have low predictive power are set to zero. Equation (1) represents the relationship between the coefficients (β) and the shrinking parameters (λ).

1 $$||y-x\beta |{|}_{2}^{2}+ \lambda ||\beta |{|}_{1}$$

The L1-regularization introduces substantial bias to the model to reduce variance and select a subset of significant predictors. This is achieved by preventing overfitting by pushing some coefficients towards zero. Therefore, the coefficients produced by this method are practically not interpretable, given that it was suppressed by the penalty, and may even undergo changes in magnitude or direction (Goeman et al., 2022). Nevertheless, even with these caveats, LASSO regression is advantageous for selecting impact variables from a pool of predictors and simplifying models (James et al., 2013).

Given previous research (Kieslinger et al., 2021; Rideout & Robb, 2020) identified children's age and gender and parents’ SES and self-efficacy as predictors for children's screen media usage, we added the net household income and the self-efficacy question score to our model. Additionally, we used the Parental Motivation for Screen Exposure factors and the BRIEF-A EFs as predictors. All numeric variables were converted to z-score before inserting them into the model.

The LASSO regression requires finding the shrinking parameter, a user-selected value. To this end, we conducted a train/test split in our sample: ~ 70% of our data (*n* = 178) to train our model and select the best-shrinking parameter based on the root mean square error (RMSE) with a leave one out cross-validation; ~ 30% of our data (*n* = 73) to test the best model and evaluate its performance by the mean absolute error (MAE), RMSE, and R2.

To do our analysis, we used R (version 4.2.2). For the LASSO regression, we used the *glmnet* package (Friedman et al., 2010). The data and the codes used in our analysis are available at https://osf.io/3g485/.

## Results

Concerning the distribution characteristics of our variables, all numerical variables demonstrated skewness and kurtosis values within the range of -2 to 2 (minimum skewness = -0.24, maximum skewness = 0.92, minimum kurtosis = -1.39, maximum kurtosis = 0.73). The QQPlots for each numerical variable utilized in this investigation are additionally accessible in the OSF repository.

The correlations between the numerical variables demonstrated that ScreenQ was significantly correlated with almost all other numeric variables. The only exception was two indexes of the parent BRIEF-A: the Self-monitor (*p* = 0.055) and the Material Organization (*p* = 0.165). All correlations with the ScreenQ were positive, except for parental self-efficacy. This indicates that higher motivations and signs of parental executive dysfunctions are associated with higher screen-based media use. The mean and standard deviation of the numeric variables and Pearson correlation coefficients of all relationships are shown in Table 2.

**Table 2:** Descriptive statistics and correlations for Study numeric variables

	*Mean*	*S. D*	*1*	*2*	*3*	*4*	*5*	*6*	*7*	*8*	*9*	*10*	*11*	*12*	*13*	*14*	*15*
*1. ScreenQ*	8.22	4.57	1.00														
*2. Parent self-efficacy*	3.74	1.09	-0.35^***^	1.00													
*3. PMSE-Q Parental need*	3.86	2.24	0.40^***^	-0.25^***^	1.00												
*4. PMSE-Q Education*	2.19	1.74	0.22^***^	-0.12	0.47^***^	1.00											
*5. PMSE-Q Children’s desire*	4.78	3.22	0.47^***^	-0.29^***^	0.71^***^	0.32^***^	1.00										
*6. PMSE-Q Behavioral Control*	3.06	1.92	0.43^***^	-0.30^***^	0.65^***^	0.36^***^	0.66^***^	1.00									
*7. BRIEF-A Inhibition*	4.06	2.96	0.17^**^	-0.08	0.21^**^	0.09	0.14^*^	0.11	1.00								
*8. BRIEF-A Cognitive Flexibility*	4.17	2.52	0.17^**^	-0.13^*^	0.14^*^	0.03	0.19^**^	0.15^*^	0.57^***^	1.00							
*9. BRIEF-A Self-monitor*	7.70	4.63	0.12	-0.06	0.15^*^	-0.02	0.19^**^	0.12	0.56^***^	0.59^***^	1.00						
*10. BRIEF-A Emotional Control*	2.92	2.35	0.14^*^	-0.09	0.07	-0.03	0.05	0.07	0.62^***^	0.52^***^	0.59^***^	1.00					
*11. BRIEF-A Initiation*	5.24	3.54	0.28^***^	-0.12	0.26^***^	0.09	0.28^***^	0.17^**^	0.67^***^	0.65^***^	0.48^***^	0.43^***^	1.00				
*12. BRIEF-A Working Memory*	5.09	3.54	0.13^*^	-0.07	0.12	0.07	0.07	0.02	0.72^***^	0.62^***^	0.42^***^	0.49^***^	0.69^***^	1.00			
*13. BRIEF-A Planning*	6.63	4.08	0.15^*^	-0.13^*^	0.17^**^	0.03	0.17^**^	0.09	0.65^***^	0.58^***^	0.39^***^	0.45^***^	0.78^***^	0.74^***^	1.00		
*14. BRIEF-A Task monitoring*	3.90	2.27	0.14^*^	-0.07	0.19^**^	0.05	0.16^*^	0.09	0.64^***^	0.58^***^	0.41^***^	0.50^***^	0.66^***^	0.77^***^	0.76^***^	1.00	
*15. BRIEF-A Material organization*	4.93	3.93	0.09	-0.08	0.15^*^	0.16^*^	0.20^**^	0.11	0.58^***^	0.41^***^	0.31^***^	0.34^***^	0.58^***^	0.53^***^	0.67^***^	0.60^***^	1.00

*S.D.* Standard Deviation, *PMSE-Q* Parental Motivation for Screen Exposure Questionnaire, *BRIEF-A* Behavior Rating Inventory of Executive Function-Adult Version

Bold values are the significant correlations

^*^indicates *p*-values < 0.05. **indicates *p*-values < 0.01. ***indicates *p*-values < 0.001

The LASSO regression model predicting young children’s screen media usage converged on the train split. From a total of 23 features, 14 were selected. In the SES, minimum wages 1 to 3, 6 to 9, and more than 20 minimum wages were not selected for the model, while parental motivation for educational purposes was also not selected. For the parental EFs, inhibition, self-monitoring, emotional control, working memory, and planning were also not selected. The coefficient of each feature is presented in Table 3 (LASSO regression). In the test split, we found satisfactory estimates: the model predicted 30.5% of the young children’s screen media usage variance.

**Table 3:** Coefficients of the LASSO regression predicting the young children’s screen media usage variance and the test split estimates

**Features**^a^	**Coefficients**
SES^b^	
*Until 1 minimum wage*	-0.02
*1–3 minimum wages*	-
*6–9 minimum wages*	-
*9–12 minimum wages*	-0.20
*12–15 minimum wages*	-0.18
*15–20 minimum wages*	-0.17
*More than 20 minimum wages*	-
Children’s gender (boy)	0.11
Children's age	0.20
Parent self-efficacy	-0.17
Parental Motivation	
*Parental need*	0.04
*Children’s desire*	0.24
*Behavioral control*	0.12
BRIEF-A	
*Cognitive Flexibility*	-0.01
*Initiation*	0.20
*Task Monitor*	-0.07
*Material Organization*	-0.05
**Performance in the test split**	**Measures**
MAE	0.63
RMSE	0.81
R^2^	0.31

^a^Only presenting the selected features by the LASSO regression, except for the SES categories. The complete list of features is described in the method section

^b^SES reference is 3 to 6 minimum wages, the category with the most participants

## Discussion

Our study explored the relationship among guardians’ EFs, motivation for their children's screen media usage, and young children's screen media usage. To do this, we employed a feature selection model. The findings provide valuable insights into the complex interplay between parental factors and children's screen media behaviors.

The results of this study highlight the significant role parents' executive functions play in shaping their children's screen media usage behaviors. The BRIEF-A allowed for a comprehensive assessment of various executive functions, including self-monitoring, task monitoring, material organization, working memory, inhibition, cognitive flexibility, planning, emotional control, and initiation. These executive functions collectively contribute to guardians' ability to control and organize their children's routines and activities, which is particularly relevant in managing screen media usage. The correlation analyses revealed a noteworthy association between parental executive dysfunction and higher screen media usage among young children. This suggests that parents with difficulties in executive functioning may find it challenging to effectively regulate and manage their children's screen time, potentially leading to higher usage than recommended. Results resonate with previous research on the potential consequences of excessive screen use on cognitive development, behavior problems, anxiety, and depression (Stiglic & Viner, 2019). Parents who want to follow guidelines such as WHO’s must have the cognitive abilities to follow them (Wilson & Gross, 2018).

The implementation of the LASSO regression model was a valuable approach to identifying the most influential predictors of young children's screen media usage. Results found by this model identified several predictors of young children’s screen media usage: SES, children's age and sex, parents’ self-efficacy, parental motivation (parental need, children’s desire, and behavioral control), and parents’ EFs (cognitive flexibility, initiate, task monitor, and material organization). These predictors collectively accounted for a substantial portion of children's screen media usage variance.

Notably, family net income underscores the role of socioeconomic factors in shaping children's screen time, reaffirming previous research (Kieslinger et al., 2021; Rideout & Robb, 2020) that links higher SES with lower screen media usage. Children in families with a net household income of 3 to 6 minimum wages were more likely to have higher screen media usage than children in families with a net household income of 9 to 20 minimum wages. No significant difference was found for children in families with incomes of 1–3 or 6–9 minimum wages, indicating that the range between 1 to 9 has the same levels of screen media usage. Furthermore, children with family net income under 1 minimum wage had lower screen media usage. This can be explained by the fact that these families may have less access to screen devices.

Children’s age and gender were also significant factors in screen media usage supported by previous findings in which boys use more screen devices than girls, while older kids engage more with screen devices (Kieslinger et al., 2021; Rideout & Robb, 2020).

Our results demonstrated that parents' self-efficacy is a critical factor influencing children's screen media behaviors. The question addressing parental self-efficacy in limiting screen use revealed that guardians' confidence in saying "no" to their child's screen-related requests was associated with decreased screen media usage. Kieslinger and colleagues (2021) also found similar results. This highlights the significance of guardians' beliefs in their ability to regulate and enforce screen time limits. Those who felt more confident in managing their children's screen media requests were likely to be more successful in adhering to recommended guidelines.

Regarding the motivation to use a screen device, our results indicated that children's desire (i.e., children asking to use it and family routine), behavioral control (i.e., rewarding the children for certain behaviors), and parental need (i.e., letting the children engage with screen devices while parents are doing other activities or engaging with screen devices as an activity between parents and children) were predictors of children screen media usage. Educational purposes were not a predictor. Our results indicated that guardians who reported using screen media as a means of behavioral control had a significant association with higher levels of children's screen media usage, implying that using screen media to reward desired behavior can inadvertently contribute to increased screen time. On the other hand, motivations related to educational purposes and children's desires were not identified as primary drivers of screen media usage; guardians may be more likely to permit screen use for non-educational and non-child-initiated reasons.

Our model also demonstrated that parental EFs were associated with children's screen media usage. Namely, cognitive flexibility, initiation, task monitoring, and material organization were all related to screen media use, while higher was related to higher screen media usage.

In conclusion, our study elevated the intricate interplay between parental factors and young children's screen media usage. By delving into the domains of executive functions, motivations, and socio-demographics, we have unearthed valuable insights into the factors influencing this digital phenomenon. In shaping the landscape of screen media engagement among young children, our findings underscore the significance of parental self-efficacy, motivations driven by children's desires and behavioral control, and specific facets of executive functions such as cognitive flexibility and task monitoring.

The findings of our study emphasize the need for a holistic understanding of parental dynamics when considering children's screen media usage. Our exploration of socio-demographic variables has illuminated the influence of factors such as family net income, children's age, and gender, further enriching the narrative of this complex issue. Our research contributes to the growing body of knowledge regarding the digital habits of young children as technology continues to evolve and intertwine with daily life. By identifying predictors and drawing attention to guardians' roles in mediating screen media experiences, our study provides a foundation for targeted interventions, educational initiatives, and parenting strategies that can contribute to healthier screen media practices among children. For example, the findings derived from our study have implications for the application of behavioral digital interventions rooted in the third wave of psychological interventions. These interventions, characterized by interactive activities, draw upon mindfulness-based techniques, ACT, and DBT to enhance cognitive, affective, attentional, motivational, and behavioral dimensions (Borghouts et al., 2021; Brigden et al., 2019; Hayes & Hofmann, 2021). Moreover, the outcomes of this investigation are in line with the recent discourse surrounding real-world data, as it encompasses behaviors of children and parents obtained through surveys. These findings contribute valuable insights for informing future healthcare decisions (Magalhães et al., 2022). Leveraging these insights, such interventions can be strategically employed to provide guidance to parents, facilitating the development of cognitive strategies aimed at mitigating excessive screen-based device usage in children.

Limitations of our study include the reliance on self-reported data and the cross-sectional design in drawing causal inferences. Future research could benefit from longitudinal approaches, objective measurements of screen time and parental EFs, and a broader range of socio-demographic factors. Longitudinal studies offer a multitude of benefits, including the potential to establish causality between screen time and developmental outcomes. By tracking children's screen habits and developmental progress over extended periods, we can determine the temporal order of events, unveil developmental trajectories, and assess cumulative effects. Moreover, this approach allows us to explore individual differences and identify long-term trends in screen media usage. Findings from longitudinal research have the potential to shape evidence-based policies, intervention strategies, and parental guidance, providing a comprehensive understanding of the interplay between screen media and child development. Furthermore, our sample consisted mostly of women, whites, and individuals with a high level of instruction. This sample is not representative since the 2010 census demonstrated that more than 50% of the nation's population was of browns or blacks (IBGE, 2012). Additionally, these results also indicate that caregiving is still carried mostly by women. The high level of instruction in our sample also denotes that the parents who answered our questionnaires probably have more access to technology, which could influence the results that we found. New studies should focus on diverse samples.

Our work contributes to the ongoing discourse on children's digital interactions. As guardians, educators, and policymakers seek effective ways to navigate the digital age, our study's insights into the intricate web of parental factors influencing young children's screen media usage offer a valuable contribution to this crucial conversation.

## Data Availability

The data and codes that support the findings of this study are openly available in Open Science Framework (OSF) at https://osf.io/3g485/.

## Abbreviations

AAP: American Academy of Pediatrics
ACT: Acceptance and Commitment Therapy
BRIEF-A: Behavior Rating Inventory of Executive Function-Adult Version
DBT: Dialectical Behavior Therapy
EF: Executive Functions
LASSO: Least Absolute Shrinkage and Selection Operator
MAE: Mean Absolute Error
RMSE: Root Mean Square Error
SD: Standard Deviation
ScreenQ: Screen-based media use in young children questionnaire
SES: Socioeconomic Status
WHO: World Health Organization
